# Effect of age on dental plaque deposition and its control by ultrasonic scaling, dental hygiene chew, and chlorhexidine (0.2%w/v) in dogs

**DOI:** 10.14202/vetworld.2019.1872-1876

**Published:** 2019-11-28

**Authors:** Nishiswapna Garanayak, Manoranjan Das, Ramesh Chandra Patra, Sangram Biswal, Susen Ku Panda

**Affiliations:** 1 Department of Clinical Medicine, Ethics and Jurisprudence, College of Veterinary Science and Animal Husbandry, Orissa University of Agriculture and Technology, Bhubaneswar, Odisha, India; 2 Department of Epidemiology and Preventive Medicine, College of Veterinary Science and Animal Husbandry, Orissa University of Agriculture and Technology, Bhubaneswar, Odisha, India; 3 Department of Veterinary Pathology, College of Veterinary Science and Animal Husbandry, Orissa University of Agriculture and Technology, Bhubaneswar, Odisha, India

**Keywords:** chlorhexidine, dental chew, dental plaque, dog, scaling

## Abstract

**Background and Aim:**

Periodontitis is the most prevalent inflammatory dental disease caused by a lack of oral hygiene measures in domestic animals. The periodontal disease complex arises as a result of bacterial biofilm deposition termed as plaque on the tooth surface. Lack of cleaning measures either mechanical or chemical credit for the condition. The present study was conducted to screen the animals for the presence of plaque deposition, gingivitis, along with various control measures for the same.

**Materials and Methods:**

Thirty-two dogs of different age groups were evaluated for the presence of plaque and gingivitis by scoring method to estimate the extent of severity. Scaling of the tooth surface was done by ultrasonic scaling machine to remove the plaques, and the animals were divided into four treatment groups to study the effects of dental hygiene chew and chlorhexidine for control of plaque.

**Results:**

Present study revealed 71.87% and 34.37% of the screened animals were having plaque deposition and varied degrees of gingivitis respectively. A positive coefficient of correlation (r) of 0.89 (p<0.05) between advancing age and plaque deposition and 0.85 (p<0.05) between age and level of gingivitis was obtained. Two groups receiving dental chew and 0.2% w/v chlorhexidine showed lower plaque deposits, and the fourth treatment group receiving both dental chew and chlorhexidine showed 100% animals remained free from fresh plaque deposits.

**Conclusion:**

The present study showed a strong positive relationship between age and plaque deposition and gingivitis. The study also showed that oral hygiene measures such as use of dental hygiene chew and chlorhexidine application can reduce plaque deposition and periodontitis in domesticated canines.

## Introduction

The oral cavity of the animal is the gateway to the digestive system. It is the foremost important organ consisting of varied degrees of anatomical structures. Periodontium is one of the major structures comprised of tooth and its supporting structures such as gingiva alveolar bone, periodontal ligament, and cementum [1]. Periodontitis is the most prevalent and unapparent inflammatory dental disease caused by bacterial plaque on the periodontium [2]. Early diagnosis of the oral infection at the stage of gingivitis and its proper treatment can reverse the damage. The disease has been reported to have many distance implications in the body organs [3,4]. The correlation between increasing age and oral disorders has already been established based on prevalence and severity studies. A large number of domestic carnivores (80%) were reported as being infected by the disease after the age of 2 years [5-7]. Newly formed calculus covered with dental plaque accounted for the advancement of the disease process leading to initiation of inflammatory cascades causing gingivitis [8].

Veterinary dentistry emphasized on importance of plaque and calculus removal from the tooth crown, gingival sulcus, and root surfaces. The removal can be done by mechanical (Brushing, scaling, ultrasonic scaling, and dental chew) and chemical (chlorhexidine gluconate, and special diet) means for the prevention and control of periodontal disease [1,4,5,9,10]. The use of dental chews of varied shapes and sizes with or without an anti-calculus agent has been demonstrated as one of the easiest methods for removal of supragingival plaque accumulation [5,8,10-12].

Although a large number of canine companions are present in the urban and semi-urban areas of Odisha, no study has been focused on their oral hygiene and status of periodontal affections. Moreover no trials have been made in the state of Odisha regarding evaluation of plaque status in domestic dogs and its removal and effective management.

This study aimed to screen the animals for the presence of plaque deposition, gingivitis, and their subsequent control by use of scaling, dental chew, and chlorhexidine treatment.

## Materials and Methods

### Ethical approval

The study was approved by Dean, College of Veterinary Science & AH, OUAT, Odisha, India. All ethical guidelines were followed during the study.

### Area of the study

The study was conducted at the Department of Clinical Medicine, Ethics and Jurisprudence, College of Veterinary Science and Animal Husbandry (AH), Orissa University of Agriculture and Technology (OUAT), Bhubaneswar. The collection of samples and selection of animals were done from Teaching Veterinary Clinical Complex of College of Veterinary Science and AH, OUAT, Bhubaneswar, private pet clinics of Bhubaneswar city and nearby areas.

### Scoring of gingivitis and plaque deposition

**Figure-1:**
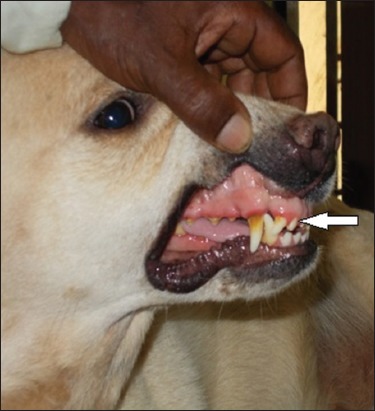
Visual assessment of plaque deposition and gingivitis (Score 2).

**Figure-2:**
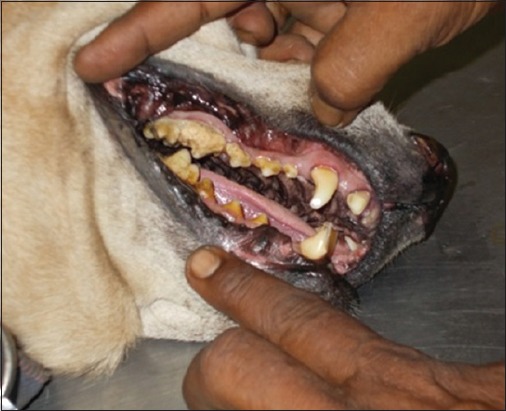
Visual assessment of plaque (plaque deposition Score 4).

The animals were screened for visible plaque deposition on the tooth surface, and scoring was done as described by Clarke *et al*. [8] with slight modification. Total visible tooth surface area (mesial, buccal, labial, lingual, and distal) covered by plaque was taken into consideration for the scoring pattern (Figures-1 and 2). Probing was done to assess tooth mobility and loosening. The details of the scoring pattern are described in Table-1.

**Table-1:** Scoring criteria for the plaque deposition.

Assigned score	Amount of plaque deposition	Coverage of plaque
0	No detectable plaque	Nil
1	Scattered plaque	<24% of the tooth surface
2	Scattered plaque	25-49% of the tooth surface
3	Less scattered	50-74% of the tooth surface
4	Almost continuous	More than 75% of the tooth surface

The severity of gingivitis was measured according to previous research methodology with slight modification [12]. Visible changes in the gingival were taken into consideration for the scoring of gingivitis. The total surface area affected and reddening of the mucosa was assessed. The details of the scoring pattern are described in Table-2.

**Table-2:** Scoring criteria for level of gingivitis.

Assigned score	Level of gingivitis
0	No gingivitis
1 (+)	Incipient gingivitis (slight red, swollen)
2 (++)	Mild gingivitis (red, swollen)
3 (+++)	Moderate gingivitis (red, swollen and occasional bleeding)
4 (++++)	Severe gingivitis (red, swollen and profuse bleeding)

### Ultrasonic scaling of the tooth surface

Plaque removal was done using Ultrasonic Scaler (Brand – DTE D1) by scaling of the teeth surface under the influence of mild anesthesia or sedation [13]. The dogs after a complete cleaning of the plaques deposits were regarded as “Clean tooth model” as described by Hennet *et al*. [14].

### Experimental design

A parallel, cross-over design clinical study on the accumulation of plaques following complete scaling of the tooth surface was done. Out of screened 32 numbers of dogs, 16 numbers of client-owned dogs were selected randomly for the study. Sixteen numbers of dogs after the preparation of clean tooth model were grouped into four trial categories randomly.

Group – 1: Animals were given normal food without any kind of dental care measures. They are regarded as negative control [8]. Group – 2: Animals were given normal food with provision of dental chew twice daily immediately after food [8]. Group – 3: Animals were given normal food and application of chlorhexidine on the buccal surface of the tooth by soaked cotton twice daily after food was done [15]. Group – 4: Animals were given normal food and provision of both dental chew and application of chlorhexidine on tooth surface done. Details of the experimental group assigned are given in Table-3.

**Table-3:** Experimental test groups.

Group–1	Normal food habit with no dental health care
Group–2	Normal food habit with the provision of dental chew BID
Group–3	Normal food habit with chlorhexidine gluconate (0.20% w/v) application BID after food
Group–4	Normal food habit with both dental chew BID and application of chlorhexidine gluconate (0.20% w/v) BID

### Dental chew

Commercially available vegetable dental chew was used for the study. The chew had ingredients such as rice flour, soy protein concentrate, vegetable glycerine, cellulose, sodium hexametaphosphate, citric acid, chlorophyll, peppermint, parsley, natural flavor, and water (calorie – 2800 kcal ME/kg, crude protein – 20%, Crude fat – 0.5-1.5%, crude fiber – 4%, and moisture – 15%). The shape of the chew was spirally twisted around the vertical axis to increase the surface area for better cleansing action.

## Results and Discussion

In 23 (71.87%) and 11 dogs (34.37%), the presence of plaque deposition and gingivitis was detected, respectively. Table-4 represents the plaque deposition score of the examined canines in relation to the age groups. This showed that with the increasing age of the animals, the plaque deposition increases on the surface of teeth. Higher incidence of periodontitis in pet dogs as high as 86% has been reported by many workers from time to time [5,6,10,16]. The outcome of the disease may have distant implications on the systemic organs [17].

**Table-4:** Age-wise visible plaque deposition score.

Age group (Years)	Number of animals				

	Score 0	Score 1	Score 2	Score 3	Score 4
0.5-1.5	10	1	Nil	Nil	Nil
2-3.5	Nil	12	1	Nil	Nil
4-6	Nil	1	2	1	Nil
8-10	Nil	Nil	Nil	2	1
>10	Nil	Nil	Nil	Nil	1

**Graph-1:**
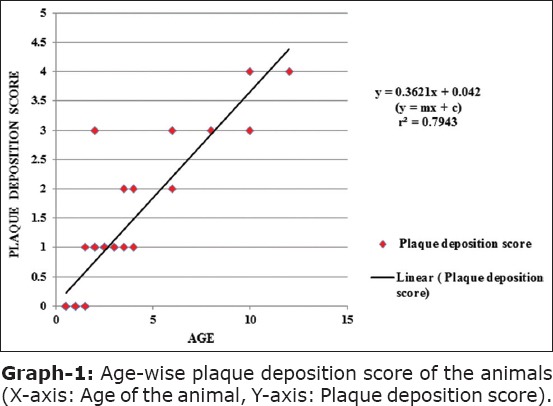
Age-wise plaque deposition score of the animals (X-axis: Age of the animal, Y-axis: Plaque deposition score).

When the plaque deposition score was plotted against the age of the screened animals, almost linear relationship (straight line) was obtained (Graph-1). The slope of the line obtained was y = 0.3621x + 0.042 with coefficient of determination (r^2^) 0.7943. This signifies the fact that with the increase in age of the animals more or less a higher plaque deposition score can always be predicted. The value of the coefficient of correlation (r) came out to be 0.89 (p<0.05), suggesting a strong positive correlation between advancing age with increased risk of plaque deposition on the surface of tooth. The Pearson coefficient of correlation (r) between age and level of gingivitis in animals was found to be 0.67 (p<0.05). Similarly, the relationship between plaque deposition score and subsequent gingivitis level came as r = 0.85 (p<0.05). It was evident from the analysis of data that the development of gingivitis was dependent on both age and plaque deposition on the tooth surface. However, gingivitis was more dependent on the amount of plaque deposition than age. This was evident from the coefficient of determination (r^2^) score of age – gingivitis (r^2^ = 0.45) and plaque deposition score – gingivitis (r^2^ = 0.73). Small dog breeds have been reported with more than 85% dentogingival alterations out of which periodontal disease, calculus, missing teeth, and abnormal attrition constitutes 60%, 61%, 33%, and 5%, respectively [5]. Similar findings in domesticated canines have been encountered by many authors [4,8,10,16].

### Ultrasonic scaling

**Figure-3:**
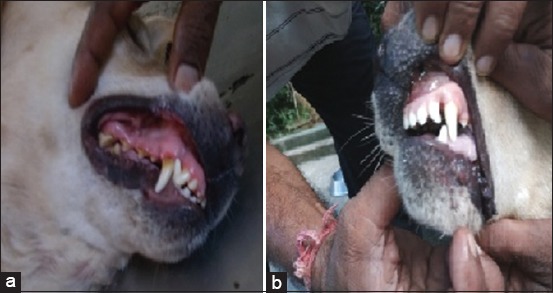
Effect of scaling of the tooth (a) before, (b) after.

After the scaling, the surface teeth were appeared to be smooth (Figure-3). The smoothness of the surface can aid in lower deposition of plaque due to the nonadherence of the bacterial biofilm. Ultrasonic scaling method was employed for removal of plaque and the animals were regarded as of clean mouth model for the experimental design. The supremacy of mechanical methods like scaling for effective removal of plaque is also commensurate with the findings of previous researchers [1,5,11,18-20].

### Experimental design

The plaque deposition pattern of the dogs under experimental design was analyzed after 28^th^ day. The detailed result of the experimental design is depicted in Table-5.

**Table-5:** Plaque deposition after the test period (28^th^ day).

Group	Dog no.	Prior plaque score	Prior gingivitis score	Percentage of teeth surface covered with plaque	Percentage of affected
Group–1	1	4	+ + +	15	50
	2	3	+	10	
	3	1	−	−	
	4	1	+	−	
Group–2	5	3	+	5	25
	6	0	−	−	
	7	1	−	−	
	8	1	−	−	
Group–3	9	1	−	−	25
	10	1	+	−	
	11	2	+	5	
	12	2	+	5	
Group–4	13	1	−	−	0
	14	0	−	−	
	15	0	−	−	
	16	3	+ +	−	

At the end of the 28^th^ day period, it was found that the two dogs (50%) of Group – 1 were shown prominent deposition of plaques on the tooth surface. In Group – 2, one dog (25%) also showed deposition of plaques. The use of dental chew for abrasive clearance of plaque has been reported by many workers [8,21-23]. Previous reports also posited that dogs receiving the daily oral chew had significantly less dental calculus (45.8%) and plaque accumulation (17.3%) compared to the dogs not receiving the same [24]. Previous experimental findings on dental chew use also reported a reduction in mean gingival score by 11.25%, affirming the findings of the present study [8]. Previous findings also reported the presence of sodium hexametaphosphate in the dental chew can aid in avoidance of plaque deposition [10].

Similarly, the observation was made in Group – 3. Two animals (50%) receiving 0.20% w/v chlorhexidine application BID after food showed fresh plaque deposition. Effectiveness of chlorhexidine for control of plaque deposition and mouth cavity infections has been documented by many workers [25-27]. Group – 4 having four animals with provision of both dental chew and chlorhexidine showed no plaque deposition after the 28^th^-day observation. The result confirmed the synergetic effects of both chemical and mechanical means of plaque removal to be most efficacious in nature.

From the result, it was observed that animals with prior high plaque deposition scores were more prone to reoccurrence of subsequent deposition. The risk can grow higher with no oral hygiene measures. The Pearson coefficient of correlation (r) between prior plaque depositions (0 day) and subsequent plaque deposition (28^th^ day) after treatments was calculated to be 0.8 (p<0.05). This result can be explained by the fact that most of the new deposition started from the interdental spaces and periodontal space where cleaning by scaling was less efficient as compared to the table surface of the teeth. Even if prophylactic measures were undertaken, plaque already deposited at difficult sites (interdental spaces and periodontal spaces) may again start new depositions. The appearance of higher amount of fresh plaques without any control measures like in the control group has been reported by many workers [8,11,12,24].

## Conclusion

The present study gathered firm evidence on the presence of periodontal disease complex in the domestic canines due to food habits and lack of oral hygiene measures. The reduced effectiveness of chlorhexidine for control of plaque deposition compared to other test groups can be attributed to the fact that the effectiveness of the chemical was transient on the tooth surface due to licking movement of the tongue. The compounding effects of both chemical and mechanical means of plaque removal in the last group were found to be most effective. This can be ascribed to the fact that both the methods in combination were effective for control of oral hygiene in domestic canines.

## Authors’ Contributions

NG conducted the study and prepared the manuscript. MD and RCP designed the study and reviewed the manuscript prepared. SB and SKP supported the study and helped in the preparation of manuscript. All authors read and approved the final draft of the manuscript.
